# Deep Sparse Autoencoder and Recursive Neural Network for EEG Emotion Recognition

**DOI:** 10.3390/e24091187

**Published:** 2022-08-25

**Authors:** Qi Li, Yunqing Liu, Yujie Shang, Qiong Zhang, Fei Yan

**Affiliations:** Department of Electronics and Information Engineering, Changchun University of Science and Technology, Changchun 130012, China

**Keywords:** EEG, emotion recognition, deep sparse autoencoder, CNN, LSTM

## Abstract

Recently, emotional electroencephalography (EEG) has been of great importance in brain–computer interfaces, and it is more urgent to realize automatic emotion recognition. The EEG signal has the disadvantages of being non-smooth, non-linear, stochastic, and susceptible to background noise. Additionally, EEG signal processing network models have the disadvantages of a large number of parameters and long training time. To address the above issues, a novel model is presented in this paper. Initially, a deep sparse autoencoder network (DSAE) was used to remove redundant information from the EEG signal and reconstruct its underlying features. Further, combining a convolutional neural network (CNN) with long short-term memory (LSTM) can extract relevant features from task-related features, mine the correlation between the 32 channels of the EEG signal, and integrate contextual information from these frames. The proposed DSAE + CNN + LSTM (DCRNN) model was experimented with on the public dataset DEAP. The classification accuracies of valence and arousal reached 76.70% and 81.43%, respectively. Meanwhile, we conducted experiments with other comparative methods to further demonstrate the effectiveness of the DCRNN method.

## 1. Introduction

Emotion recognition is one of the most core and basic issues of affective computing [1]. With the development and application of computer technology, emotion recognition has played a huge role in promoting medical treatment, education, elderly care, criminal investigation, and human–computer interaction. [2] Currently, automatic emotion recognition includes both discrete and continuous emotion models for emotion recognition from physiological and non-physiological signals. Non-physiological signals such as text, language, and facial expressions are highly subjective [3]. Wearable and non-invasive physiological signals have the advantage of real time and objectivity [4]. Compared with the discrete emotional model, the continuous dimension emotional model can accurately describe the emotional state of people and fit the real feelings of people, which has become the goal of researchers in emotion recognition.

In previous studies, many researchers adopted traditional machine learning algorithms for emotion recognition. Support vector machines (SVM) and K-nearest neighbors (KNN) are widely used for feature classification in sentiment classification tasks [5,6,7,8]. Kumar et al. [9] used a linear kernel least squares support vector machine and back-propagation artificial neural network to perform binary emotion recognition on the valence and arousal models, and the accuracy rates reached 61.17% and 64.84%, respectively. Wang et al. [10] used a bidirectional long short-term memory (BLSTM) network for improved experiments and achieved better recognition accuracy in the SEED dataset [11]. On the SVM classifier, the effective feature screening and karyotype classifier were used to classify sentiment, and the valence and arousal accuracies of the SVM classifier were 73.06% and 73.14%, respectively [12].

At present, emotion recognition based on machine learning has achieved certain results. Islam et al. provided a critical review and summary of the recently published literature that clearly demonstrates the development of state-of-the-art emotion recognition [13]. However, due to the relative simplicity of traditional machine learning methods and poor generalization ability, many researchers have introduced deep learning into emotion recognition research and achieved certain results. A deep learning-based neural network model was proposed by Islam et al. One-dimensional EEG data were converted into feature images with Pearson correlation coefficients, and then convolutional neural networks were used for emotion recognition. The method alleviates the workload of performing feature extraction manually but still requires contributions in terms of important feature extraction as well as network optimization [14]. Jirayucharoensak et al. [15] built a deep learning network by stacking autoencoders to achieve hierarchical feature learning of EEG signals. Principal component analysis (PCA) was applied to extract the most important part of the initial input features, and the final recognition accuracy was 53.42% and 52.05%, respectively. The important information extracted by the PCA method still included unimportant and redundant information that does not adequately extract the emotional features of the EEG signal. Li et al. [16] extracted PSD features from a multi-channel EEG, constructed multi-dimensional feature images, and then adopted CNN, LSTM, and a recurrent neural network to construct a hybrid neural network model, CLRNN. The average sentiment classification accuracy for each subject in the DEAP dataset reached 75.21%. For the direct use of convolutional neural networks to classify EEG signals, there are disadvantages such as high computational effort and a long training time. On this basis, autoencoding technology has been widely used in biological information processing, especially for the reconstruction and feature extraction of high-dimensional signals. Zhang et al. [17] detected mental activity by building a sparse autoencoder network to extract the emotional features of the respiration signal, and he valence and arousal classification accuracies on DEAP were 73.06% and 80.78%, respectively. Not only respiratory signals but also EEG signals contain a rich emotional profile that can detect mental activity. Xing et al. [18] used a stacked autoencoder to build a linear EEG signal mixture model and finally chose LSTM-RNN as the emotion classifier. The valence and arousal classification accuracies on DEAP were 81.10% and 74.38%, respectively. Construction of network models still need to be improved in terms of computational effort and training time.

Although methods for EEG emotion recognition emerge in an endless stream, there are still two important challenges that need to be studied.

**Figure 1:**
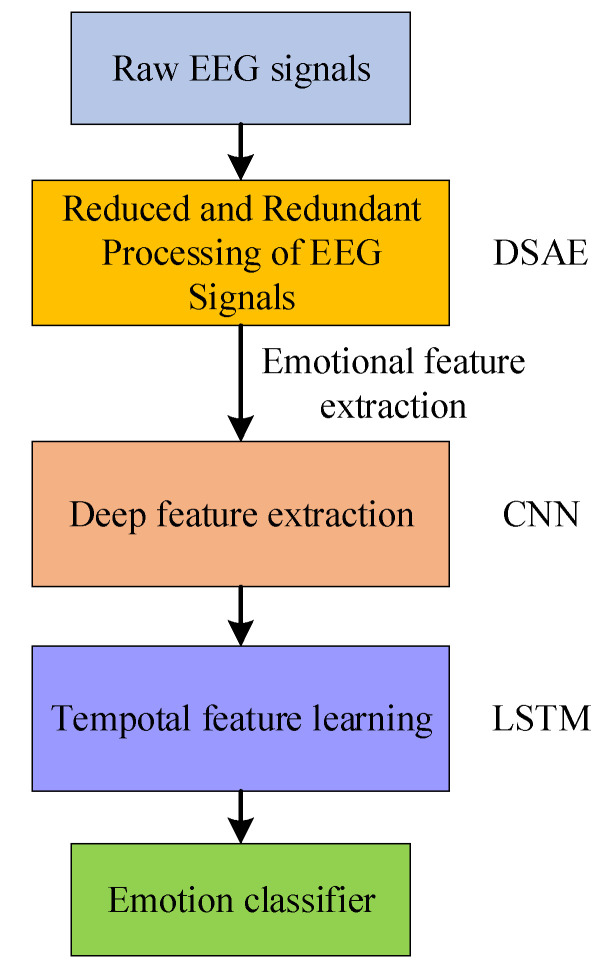
The algorithm and flowchart of the whole work.

1. To deal with the disadvantages of being non-smooth, non-linear, random, and susceptible to the background noise of EEG signals, we proposed a method for downscaling and removing redundant information from source EEG signals using deep sparse autoencoding networks.

2. Among the disadvantages of using convolutional neural networks directly for the classification of EEG signals there are disadvantages such as a large number of parameters and a long training time. We proposed a hybrid neural network approach that reduces computational parameters and training time, while better exploiting the correlation between the 32 channels of the EEG signal and improving the accuracy of emotion recognition. The flow chart for this is shown in Figure 1.

## 2. Methods

In this section, we present the rationale for the individual modules that make up the framework of this paper and then detail the network structure used in this paper.

### 2.1. Sparse Autoencoder (SAE)

**Figure 2:**
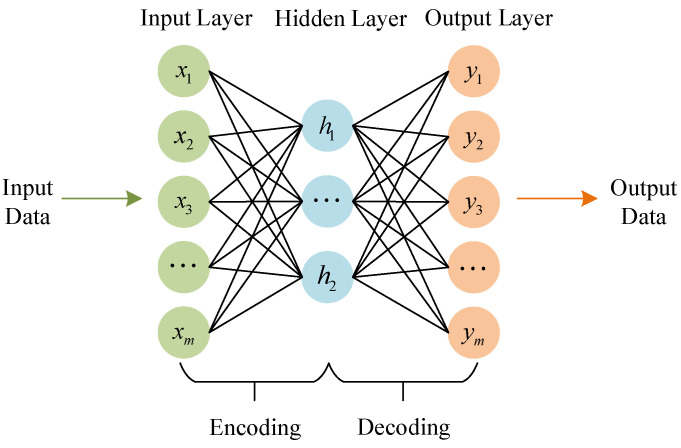
Autoencoding neural network architecture.

Autoencoder (AE) is a neural network that equalizes the output value with the input value through a back-propagation algorithm [19]. First, the input is compressed into a possible spatial representation, which is then used to reconstruct the output. The autoencoding neural network architecture is shown in Figure 2.

The autoencoder consists of two parts, encoded and decoded, which are divided into three layers, namely, the input layer x, the hidden layer h, and the output layer y. The cost function used in conventional AE is the mean square error (MSE), as shown in Equation (1).

(1) $$J_{\mathrm{AEcost}\;}(W)=J_{\mathrm{MSE}}(W)=\frac{1}{m}\sum_{i=1}^{m}\left[\frac{1}{2}{\left\|y_{i}-x_{i}\right\|}^{2}\right]$$

where m is the number of samples, $x_{i}$ is the input vector, $y_{i}$ is the output vector, and W is the set of all parameters in the network.

**Figure 3:**
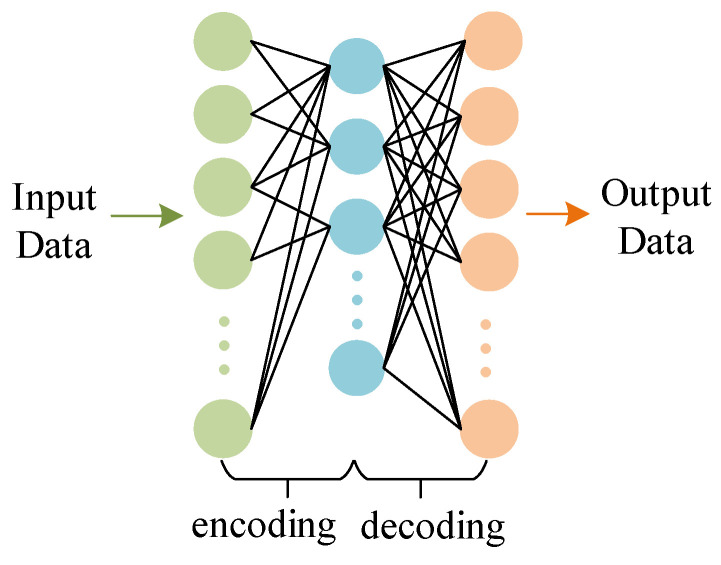
Sparse autoencoder neural network architecture.

In order to overcome the defect of redundancy in the abstract features learned by the autoencoder, based on the autoencoder, the regularization limit of L1 is increased to obtain a sparse autoencoder. SAE employs sparse constraints to eliminate feature redundancy during encoding and decoding. It increases the constraints on the response of each hidden layer, so that most neurons are “inhibited” and only a few “excited”, which is reflected in the model by adding sparse constraints to the cost function. The principle of the SAE network is shown in Figure 3. In the cost function of the autoencoder, add the following sparse constraints:

(2) $$J_{\mathrm{SAEcost}}(W)=J_{\mathrm{MSE}}(W)+J_{\mathrm{Sparse}\;}(W)$$

(3) $$J_{\mathrm{Sparse}\;}(W)=\beta \sum_{i=1}^{2}KL\left(\rho |\;\rho_{j}\right)$$

(4) $$KL\left(\rho |\;\rho_{j}\right)=\rho \log\frac{\rho }{\rho_{j}}+(1-\rho )\log\frac{1-\rho }{1-\rho_{j}}$$

where $\rho_{j}$ is the average activation of the hidden layer unit neurons, ρ is the sparsity constraint level, β is the weight of the sparsity penalty term, and KL is the divergence, which ensures the sparsity of neurons in a hidden layer. As shown in Equation (4), the closer ρ and $\rho_{j}$ are to each other, the smaller the cost function is.

### 2.2. Hybrid Neural Network Methods

**Figure 4:**
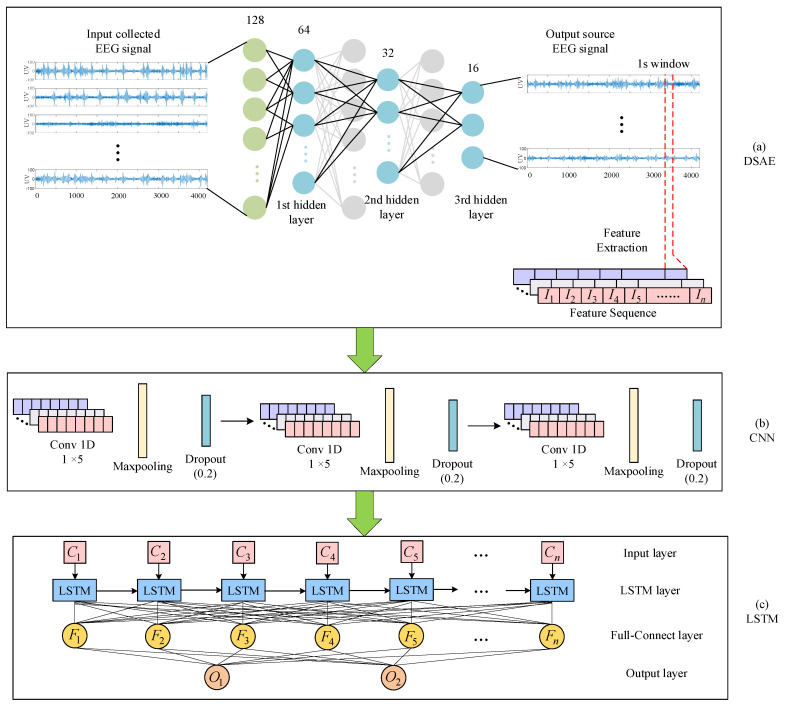
Combining deep sparse autoencoders (DSAE) with hybrid deep neural network architecture for emotion recognition with CNN and LSTM.

During the acquisition process of EEG signals, it is easy to obtain interference from various factors such as the environment and human emotional fluctuations. Therefore, various kinds of noise may be mixed in the EEG signal, which undoubtedly affects the desired brain patterns and experimental results. In addition, when EEG emotion classification is performed, there are problems such as the insufficient extraction of EEG emotion features, and it is easy to ignore EEG timing information. In order to solve the above problems, we proposed a neural network learning framework, as shown in Figure 4.

Firstly, a deep sparse autoencoding (DSAE) algorithm was used to reduce the dimensionality of the EEG to obtain an EEG with redundant information removed. The deep sparse autoencoder network was composed of multiple sparse autoencoder networks stacked, as shown in Figure 4a. The sparse autoencoder drew on the neuron excitability mechanism of the brain. After encoding, the original data were decoded to the greatest extent possible. At the same time, it also had the advantages of a fast convergence speed and training did not easily fall into the local minima. The deep sparse autoencoding network contained three hidden layers. After the encoding was completed, the brain power signal was extracted from the last hidden layer. The original DEAP EEG signal was collected at 8064 samples in 1 min, with 7680 samples after removing the 3-s baseline signal. A 1-s window was applied to the EEG source signal, dividing the signal into 128 frames. After the signal was framed, EEG emotional features were extracted from each frame by a feature extraction method and arranged into a 128-frame feature sequence.

**Figure 5:**
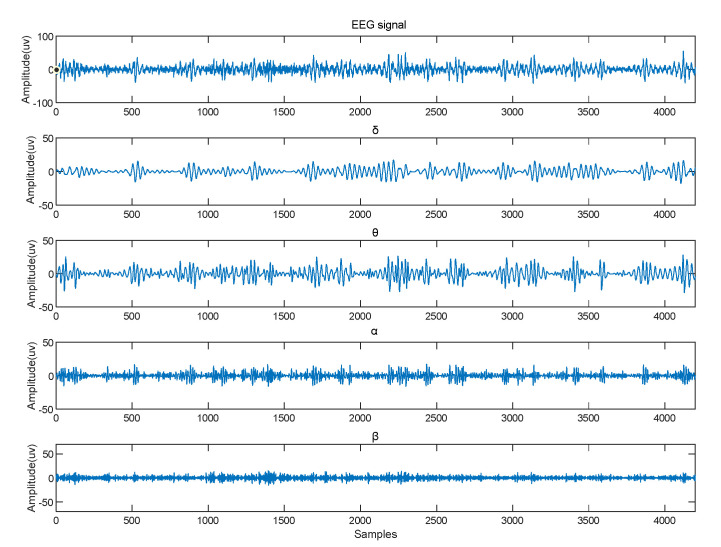
EEG raw signal and brain wave in four frequency bands.

**Figure 6:**
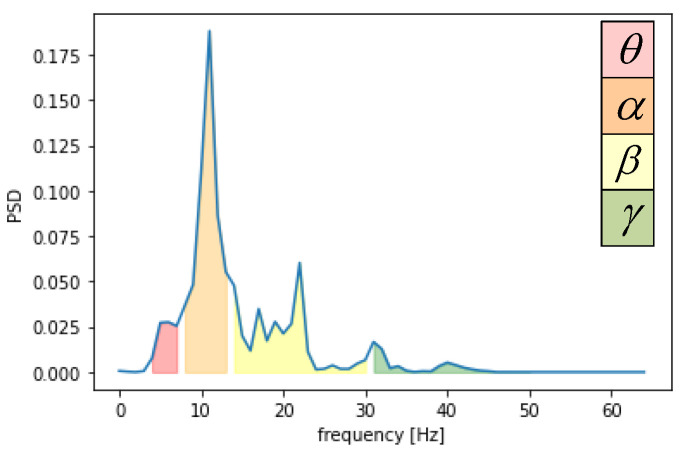
PSD value according to four bands.

Secondly, the condition of the brain changed, which was determined by rhythmic signals from various parts of the brain. EEG signals were divided into θ (4–7 Hz), α (8–13 Hz), β (14–30 Hz), and γ (31–50 Hz) according to the frequency range. The EEG raw signal in the DEAP dataset is shown in Figure 5, along with the four frequency bands. We applied a “hanning window” to each EEG channel and used the Welch method [20] to calculate the PSD characteristics. The PSD values calculated from the four bands of the signal are shown in Figure 6. The PSD feature sequence was used as the input of the neural network, as shown in Figure 4b. For the CNN part, the emotional features of the EEG signal for each channel were extracted automatically using a one-dimensional convolutional neural network. We set up three convolutional layers, each followed by a maximum pooling layer and a dropout layer. Based on the input feature sequences, we chose a suitable convolutional kernel size of 1 × 5 and a step size of 1. This allowed us to fully traverse each EEG emotion feature. An appropriate kernel size not only extracts the emotional features adequately but also reduces the number of parameters generated during the training process. The convolutional layer was followed by a rectified linear unit (ReLU) activation function to incorporate non-linear factors so that the output of some of the neurons in the network was 0 after training, providing a moderate degree of sparsity and accelerating convergence of the network. It also reduced the interdependence of the parameters and avoided the overfitting problem of the model, thus improving the generalization ability of the model.

Finally, since the EEG signal is a complex time series, to perform the emotion classification of EEG signals, the emotional state determination was determined by the characteristics of the EEG sequence. Although some useful emotional information has a long interval, it still needs to be retained; so, the selection of classification algorithms must consider the influence of time series on features. The neurons of the long-term memory recurrent neural network have the advantage of long-term memory, which can retain the long-term and short-term emotional information in the EEG signal, which is conducive to emotion recognition. Because of the characteristics of the LSTM gating unit, the network had the function of preventing gradient disappearance (and explosion) and was more suitable for the training and classification of long time series. As shown in Figure 4c, the emotional EEG feature sequence generated by the neural network was input into the long- and short-term memory recurrent network, and the supervised learning model was trained, cross-validated, and tested. First, context-relevant information was mined in EEG signal sequences using LSTM techniques [21]. The second layer was a complete connection layer, which played an important function of classification. In the LSTM layer, 128 LSTM units were used, corresponding to 128 frame features, respectively. At a fully connected level, the number of connected units was the same. Finally, we used sigmoid to launch functions at the output level. The classification and recognition results were output in the two emotional dimensions of valence and arousal. In the classification algorithm, the mini-batch gradient optimal algorithm and an SE loss function were used. To prevent overfitting, we added dropout and fully connected layers after the LSTM layer, respectively.

## 3. Experiments and Results

In this section, the paper will introduce the dataset and the processing of emotion labels and then report and discuss the results of the proposed method on the dataset as well as the analysis of comparative experimental results with other methods.

### 3.1. Datasets and Emotion Label Processing

**Figure 7:**
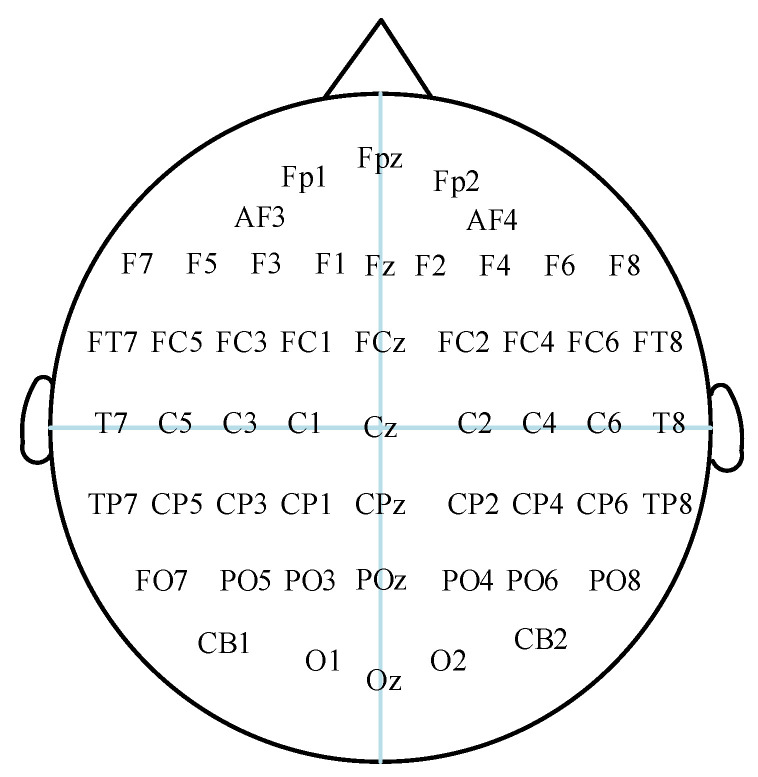
EEG electrodes’ position in DEAP dataset.

DEAP data [22] contains 32 subjects. Each subject has 32 channels of EEG signals and 8 channels of peripheral physiological signals. The 32-channel EEG signal was used as the experimental data for this paper. The electrode distribution positions are shown in Figure 7. The EEG signal was first sampled at a sampling frequency of 512 Hz; then, the sampling rate was reduced to 128 Hz and filtered by a bandpass filter of 4.0~45.0 Hz to remove electro-oculogram (EOG) artifacts. Each subject watched 40 1-min, emotional, music videos. After each video, the subjects were asked to self-assess through the SAM questionnaire on four dimensions of emotion: valence, arousal, dominance, and liking. The scale is based on a 9-point scale, with low scores indicating weakness and high scores indicating strength. The content of the DEAP dataset is shown in Table 1.

**Figure 8:**
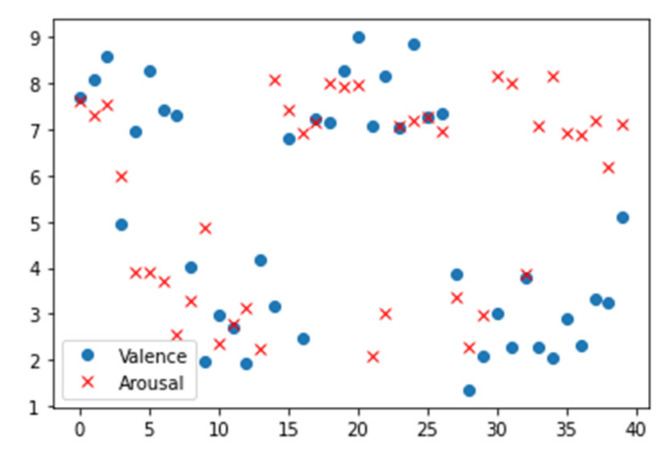
DEAP dataset sentiment label classification.

Different from the usual discrete emotion models, the DEAP dataset adopts a continuous dimension emotion model to classify emotion states. The sentiment label classification of the DEAP dataset is shown in Figure 8. In this experiment, only two dimensions of arousal and potency were selected for testing. On the valence dimension, two affective thresholds of 4.5 and 5.5 were used to classify affective states into two categories, low valence (LV < 4.5) and high valence (HV > 5.5). In terms of arousal, the same threshold was used to divide emotions into low arousal (LA < 4.5) and high arousal (HV > 5.5). Under this emotion threshold division, the numbers of high and low valence samples in the DEAP dataset were 587 and 472, respectively, and, in terms of arousal, the numbers of high and low arousal samples were 622 and 464, respectively.

### 3.2. Experiment Setup

The model was implemented with a TensorFlow framework and trained on a Nvidia Quadro P5000 GPU. We used a 10-fold cross-validation method for experimental validation [23]. We used a stochastic gradient descent (SGD) as the optimizer for optimizing the objective function with appropriate smooth features. MSE was used as the loss function.

### 3.3. Emotion Recognition Results

**Figure 9:**
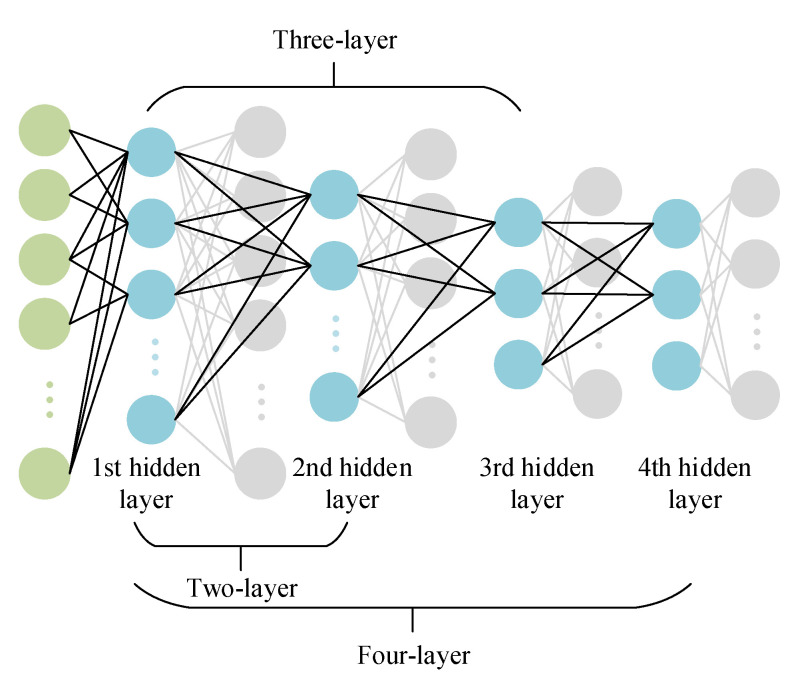
The structure of various hidden layers.

In order to explore the DSAE structure suitable for EEG data classification, we designed a DSAE with two-layer, three-layer, and four-layer structures when constructing a deep sparse autoencoder network. The structure of various hidden layers is shown in Figure 9. The number of nodes in the hidden layer was set according to the number of nodes in the input and output layers. Since the sparse self-coding network was to compress and downscale the EEG signal to obtain the most representative emotional information in the EEG signal, the input layer of the SAE was 128 frames of the EEG signal; so, the number of nodes in the hidden layer should not be larger than 128. For the layer setting of the hidden layer, we performed a comparison experiment of two-, three-, and four- layer structures. According to the dimension of the input EEG data, for the DSAE containing two hidden layers, the number of neurons in each layer was set to 64 and 16. First, a layer of the SAE was constructed. After pre-training the SAE, its weights were saved. Then, the vector A composed of the hidden activation values of the first layer was used as the input of the second layer, and the weights of the second layer were obtained by continuing training and saved as the input value of the next CNN-LSTM classification training. Then, we used the EEG data for overall training and fine-tuning the entire network. For the DSAE with three hidden layers, the number of neurons in each layer was set to 64, 32, and 16. According to the above principle, it was obtained based on the two-layer DSAE network training. For the DSAE with four hidden layers, the number of neurons in each layer was obtained. The number of neurons was set to 96, 64, 32, and 16, and the same method was used for training.

**Figure 10:**
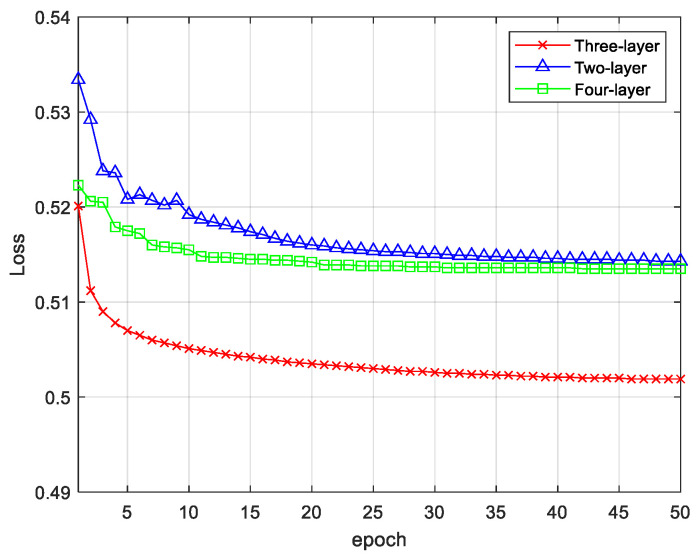
Comparison of DSAE error.

**Figure 11:**
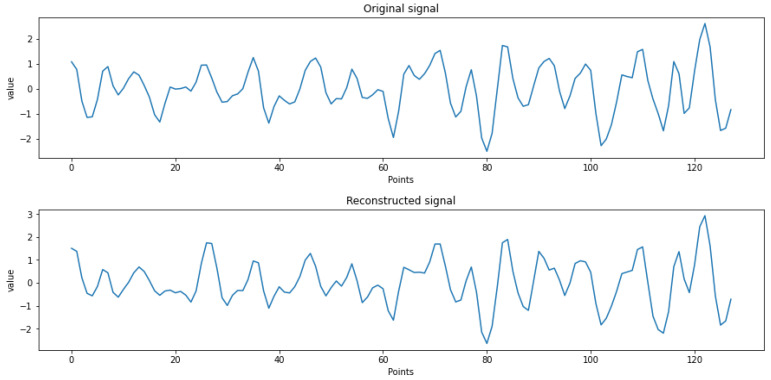
Original EEG signal and reconstructed signal loss of three structures.

The loss values of the comparative experiments of the three-structure DSAE networks are shown in Figure 10. The loss rate on the DSAE with a two-layer structure stabilized after 50 iterations. After 50 iterations, the classification loss rate of the three-layer DSAE dropped from the initial 0.52 to 0.50, which was about 0.01 lower than that of the two-layer DSAE. The DSAE loss rate of the four-layer structure was finally 0.51, which was slightly higher than that of the three-layer structure. Through the loss value graph, it can be seen that the DSAE with the lowest loss rate was the three-layer structure and it can also be seen that the DSAE three-layer structure reached the steady state more quickly. To verify the reconstruction ability of the autoencoder, we plotted the original EEG signal; the reconstructed signal is shown in Figure 11. We can see that the reconstructed signal maintained the largest features of the original signal.

In this work, the two signals were compared in terms of two important parameters, the mean square error and the signal-to-noise ratio, as shown in Table 2. The mean squared error (MSE) is a measure that reflects the difference between the actual measured value and the true value. The smaller the MSE value is, the closer the predicted value is to the true value, indicating that the signal contains less noise and the reconstruction quality is high.

(5) $$\mathrm{MSE}=\frac{1}{N}\sum_{j=1}^{N}{\left[x(j)-\hat{x}(j)\right]}^{2}$$

The signal-to-noise ratio (*SNR*) is one of the commonly used measures of signal quality. The greater the signal-to-noise ratio is, the closer the signal is to the original signal, with all other criteria being equal.

(6) $$SNR=101g\frac{\sum_{j=1}^{N}x^{2}(j)}{\sum_{j=1}^{N}{\left[x(j)-\hat{x}(j)\right]}^{2}}$$

where x(j) and $\hat{x}(j)$ denote the original signal and the reconstructed signal.

After the training data were encoded by the deep sparse autoencoding model, the encoded data were transformed to extract the PSD feature of the signal, which was used as the input for the emotion recognition and classification of the CNN + LSTM framework in this work. Classification training was performed on the DEAP dataset; the accuracy of valence was achieved in 76.70%, and arousal was achieved in 81.43%.

**Figure 12:**
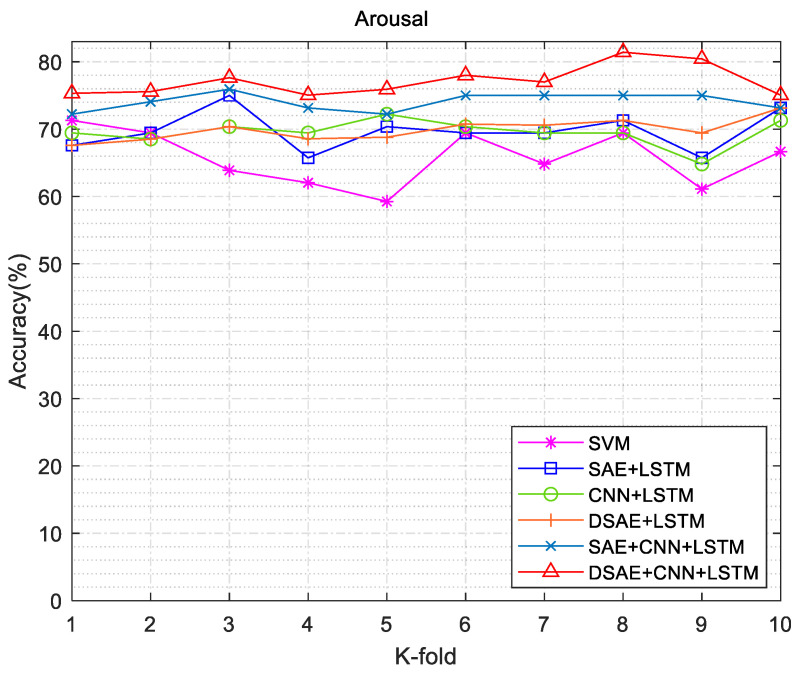
Arousal accuracy results of different experimental validation methods.

**Figure 13:**
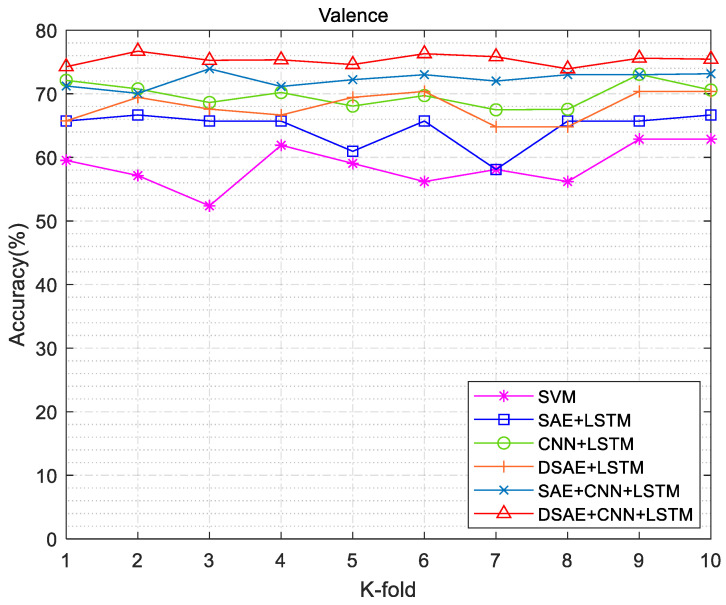
Valence accuracy results of different experimental validation methods.

We conducted exhaustive experiments to demonstrate the state of the art of DCRNN in sentiment classification. In the comparison experiments, we used SVM as the classifier to set the baseline accuracy. The feature extraction method was changed. For each channel of EEG data, the Welch method was used to calculate the PSD values; then, the frequency band power (FBP) for the four different bands was calculated using integration. The extracted features were fed into the SVM model, whose “RBF” kernel allowed for better differentiation between the different categories. All hyperparameters were left at their default values. The method in this work was mainly divided into two aspects: (1) The encoding and decoding process of DSAE was used to reduce the dimension of EEG signals and remove redundant information. (2) We used the CNN + LSTM combined neural network to classify the emotional features of the EEG signals after dimension reduction. The specific experimental combination operations are shown in Table 3. The 10-fold cross-validation experiments were used in the experimental process, and the SVM method was used as the basic method for comparison. The valence and arousal accuracy results of different experimental validation methods are shown in Figure 12 and Figure 13. DSAE + CNN + LSTM had a better sentiment classification effect.

The most common evaluation metric in classification problems is accuracy (ACC), which directly reflects the proportion of correct scores and is very simple to calculate. However, in practical classification problems, there may be some differences in the amount of data in each category, which may result in a high overall accuracy (ACC) but poor classification results in some categories. In this case, the ACC alone could not be used as an evaluation criterion for the model. For this reason, the variance and kappa coefficients of the classification accuracy of the model were calculated to measure the goodness of the classification model. The results are shown in Table 2. The kappa was used for consistency testing and is calculated as:

(7) $$k=\frac{p_{0}-p_{e}}{1-p_{e}}$$

where $p_{0}$ is the sum of the number of correctly classified samples in each category divided by the total, which is the overall classification recognition rate, and $p_{e}$ is the probability that the expected result is the same as the true result.

**Figure 14:**
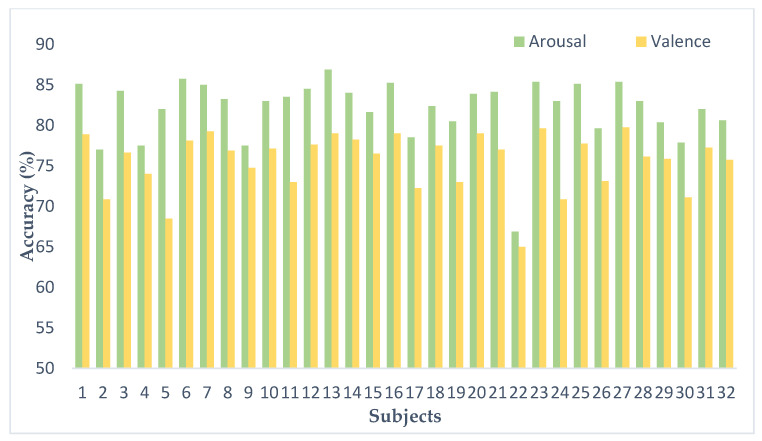
Classification accuracy of each subject on the DEAP dataset.

Classification accuracy was recorded for each subject. As can be seen in Figure 14, there were differences in classification accuracy for different subjects. In terms of the arousal classification, the average correct rate for the 32 subjects was 81.4%, with a classification accuracy of 86.88% (the highest) for subject 13 and 66.87% (the lowest) for subject 22. In terms of valence, the average correct rate for the 32 subjects was 76.70%; for subject 23, classification accuracy reached 79.63% (highest) and for subject 22 accuracy was 65% (lowest). This reflects individual variability. It is noteworthy that the validity and arousal accuracy for subject 22 was 66.87% and 79.63%, respectively, which were lower than the other subjects. The reason for this may be that the subjects lacked attention during the experiment or did not report well on the extent of subjective feelings after the watching the video.

**Figure 15:**
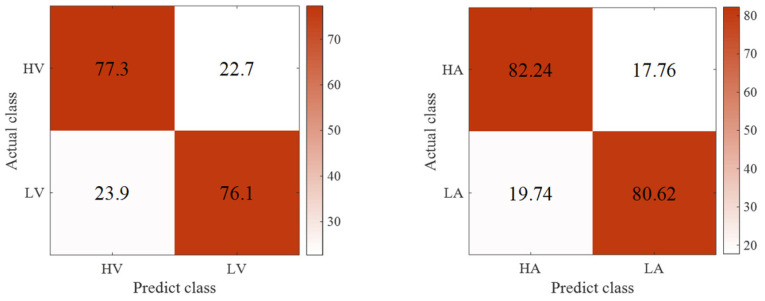
Confusion matrix: (**left**) valence, (**right**) arousal.

The confusion matrix is shown in Figure 15. Table 4 details the precision, specificity, and sensitivity metrics of the model in this work. The advancedness of the model in this paper in EEG signal emotion recognition was fully demonstrated.

In Table 5, we further list the related works with a high citation rate in recent years and the corresponding performance obtained. We used the same dataset and EEG signals in our comparison experiments with other methods. Ding et al. [24] proposed a multiscale convolutional neural network to achieve the classification of emotions in EEG by learning discriminative representations on temporal and channel dimensions. Ullah et al. [25] proposed an ensemble learning algorithm that uses a kernel representation to describe the EEG channel and performs internal emotion recognition by solving an objective function. Li et al. [26] converted one-dimensional EEG sequences into a grid-like framework by wavelets and scale maps and designed a hybrid deep learning model to identify emotions. Xing et al. [18] proposed a novel, emotion-based, multi-channel EEG hybrid mode, and emotion a mode structure was established. The models proposed in this work all showed good average classification accuracy. The method in this paper decreases the training time in network training compared to other methods. Additionally, the network runs generated a smaller number of parameters and reduced the complexity of the model. Adequate extraction of key information and the ability to identify channel relevance are key techniques that need to be addressed in network learning, and the method in this paper can address this challenge to some extent.

## 4. Conclusions

This paper proposed a novel EEG emotion recognition model. Firstly, based on the DSAE model, it was used for the decomposition of EEG signals and the extraction of channel correlation. Choosing the appropriate number of SAE layers not only improved the computational efficiency of feature extraction but also enhanced the accuracy of sentiment recognition. Then, we used a CNN + LSTM combined network model to learn and process the contextual correlation of EEG time series features to improve the recognition accuracy. The comparative results in our experiments demonstrated the effectiveness of our framework, achieving 81.43% accuracy in arousal and 76.7% in valence in the sentiment recognition task for DEAP data. Automated fast and accurate emotion recognition is important in real-time emotion monitoring scenarios. We, therefore, wish to enhance our paper by constructing our own dataset and validating the effectiveness of the methods in this paper in real-world scenarios.

## Figures and Tables

**Table 1:** DEAP dataset content.

Name	Size	Contents
Data	40 × 40 × 8064	video × channel × data
Labels	40 × 4	video × label (valence, arousal, dominance, liking)

**Table 2:** Important parameters of the original and reconstructed signals.

Signals	MSE	SNR
Original signal	0.020	32.16
Reconstructed signal	0.018	31.05

**Table 3:** Ablation experiments for combination model comparison on DEAP.

Base Model	Combined Validation Model	Accuracy (%)		Kappa	$\mathrm{Variance}\;(\times 10^{-2})$
		Arousal	Valence		
SVM	-	71.30	62.90	0.66	0.16
Without SAE	CNN + LSTM	72.23	73.07	0.67	0.27
SAE	SAE + LSTM	75	66.67	0.72	0.18
	SAE + CNN + LSTM	75.93	73.15	0.79	0.12
DSAE	DSAE + LSTM	73.14	70.37	0.76	0.08
	DSAE + CNN + LSTM	81.43	76.70	0.93	0.05

**Table 4:** Classification outcomes of our model.

Valence/Arousal	Class	Precision (%)	Sensitive (%)	Specificity (%)
**Valence**	High	79.2	73.1	76.2
	Low	74.0	79.5	74.9
**Arousal**	High	84.7	78.7	77.9
	Low	79.6	85.3	78.5

**Table 5:** Compared with the results reported in the existing literature on DEAP.

Classification Methods	Features	Arousal (%)	Valence (%)	Time Cost (s)	Parameters
Ding et al. [24]	Temporal dynamics + spatial asymmetry	61.57	59.14	1360	41,654
Ullah et al. [25]	PCA	70.10	77.40	753	12,563
Li et al. [26]	CWT	74.12	72.60	630	10,056
Xing et al. [18]	FBP	74.38	81.10	300	9443
DSAE + CNN + LSTM (DCRNN)	PSD	81.43	76.70	260	8384

## Data Availability

Data are available in a publicly accessible repository that does not issue DOIs. These data can be found at the following address: http://www.eecs.qmul.ac.uk/mmv/ datasets/deap/index.html (accessed on 20 July 2022).
